# The Mechanisms Underlying Vertical Artifacts in Lung Ultrasound and Their Proper Utilization for the Evaluation of Cardiogenic Pulmonary Edema

**DOI:** 10.3390/diagnostics12020252

**Published:** 2022-01-20

**Authors:** Toru Kameda, Naohisa Kamiyama, Nobuyuki Taniguchi

**Affiliations:** 1 Department of Clinical Laboratory Medicine, Jichi Medical University, Shimotsuke 329-0498, Japan; taniguch@jichi.ac.jp; 2 Ultrasound Division, GE Healthcare Japan, Hino 191-8503, Japan; naohisa.kamiyama@ge.com

**Keywords:** lung ultrasound, cardiogenic pulmonary edema, B-line, vertical artifact, spatial compound imaging, focal point

## Abstract

The recent advances in lung ultrasound for the diagnosis of cardiogenic pulmonary edema are outstanding; however, the mechanism of vertical artifacts known as B-lines used for the diagnosis has not yet been fully elucidated. The theory of “acoustic trap” is useful when considering the generation of vertical artifacts. Basic research in several studies supports the theory. Published studies with pilot experiments indicate that clarification of the relationship between the length and intensity of vertical artifacts and physical or acoustic composition of sources may be useful for differentiating cardiogenic pulmonary edema from lung diseases. There is no international consensus with regard to the optimal settings of ultrasound machines even though their contribution to the configuration of vertical artifacts is evident. In the clinical setting, the configuration is detrimentally affected by the use of spatial compound imaging, the placement of the focal point at a deep level, and the use of multiple focus. Simple educational materials using a glass microscope slide also show the non-negligible impact of the ultrasound machine settings on the morphology of vertical artifacts.

## 1. Introduction

In lung ultrasound, the presence and severity of pulmonary edema are evaluated with vertical artifacts known as B-lines. In an international consensus statement published in 2012, B-lines were defined as discrete, laser-like vertical hyperechoic artifacts that arise from the pleural line and extend to the bottom of the screen without fading [1]. The term “multiple B-lines” refers to the presence of three or more B-lines in a longitudinal plane between two ribs. In patients with cardiogenic pulmonary edema, multiple B-lines are usually distributed bilaterally and diffusely [1,2]. A multicenter, prospective study found that the implementation of lung ultrasound in addition to the initial conventional assessment improved the diagnostic accuracy for cardiogenic pulmonary edema [3]. A systematic review and meta-analysis demonstrated that lung ultrasound has higher sensitivity than, and similar specificity to, chest X-ray in the diagnosis of cardiogenic pulmonary edema [4]. Lung ultrasound can also drastically contribute to reducing the time spent on the diagnosis [5]. In addition, the number and spatial extent of B-lines allow the assessment of the severity of pulmonary edema or a semi-quantitative estimation of extravascular lung water [6].

Recent advances in lung ultrasound for the diagnosis of cardiogenic pulmonary edema have been outstanding, as mentioned above. However, the mechanisms underlying the vertical artifacts in cardiogenic pulmonary edema have not been fully elucidated. Furthermore, there is no international consensus with regard to the optimal settings of ultrasound machines even though their contribution to the configuration of vertical artifacts is evident [7,8,9]. They may affect the diagnostic accuracy inappropriately [9]. In this article, we review the clinical knowledge and basic research on the generation of vertical artifacts. We then demonstrate how the machine settings impact the configuration of the vertical artifacts.

## 2. Generation of Vertical Artifacts

### 2.1. Clinical Implications

In cardiogenic pulmonary edema, a rapid increase in hydrostatic pressure in the pulmonary capillaries leads to increased fluid transfer into the interstitium and alveolar spaces. High capillary pressures can also cause lung injury and barrier disruption which increases permeability and fluid transfer [10].

Chest CT is not a standard imaging modality to diagnose cardiogenic pulmonary edema; however, it is very useful for grasping the distribution of edema in the lung tissue. The findings of interstitial pulmonary edema are ground-glass opacities and interlobular septal and bronchovascular thickening. Alveolar edema appears as airspace consolidation, in addition to the above findings [11,12].

Lichtenstein et al. compared ultrasound images with CT images and indicated that B-lines originate from the thickening of the sub-pleural interlobular septa and ground-glass opacities [13]. Many researchers then reported the association of cardiogenic pulmonary edema and lung diseases with B-lines in their observational studies. Now, B-lines are thought to be generated when the air content decreases and lung density increases due to transudate, exudate, blood, collagen, or hyper-cellularity in the subpleural space [14]. However, the sonographic–pathologic correlation in B-lines and their origin in the subpleural space have not yet been fully elucidated with a more scientific method [7]. On top of that, B-lines based on the current definition [1] are not specific for pulmonary edema; therefore, clinicians have to consider their distribution in addition to history and physical examination findings for the diagnosis.

### 2.2. The Theory of Acoustic Trap

Soldati et al. introduced the interesting theory of “acoustic trap” in the generation of vertical artifacts, including B-lines [15]. An acoustic trap corresponds to a small volume of fluid in cardiogenic pulmonary edema, inflammatory changes in pulmonary diseases, surrounded by aerated alveoli with an acoustic channel on top of the trap at the pleural line. Once an ultrasound beam enters the trap through the channels, it is trapped and reflected by the wall of aerated alveoli multiple times with scattering. The reflection and scattering phenomena act as successive ultrasound sources, with the trapped energy radiated to the transducer little by little. With a larger channel, the ultrasound energy can escape more easily, and opportunities for reflection consequently decrease [7,16].

### 2.3. Our Basic Research Supporting the Theory

We developed simple experimental models that generate vertical artifacts [7,8]. In one of our previous studies, a long vertical artifact was generated by a spindle-shaped juice sack and a string-shaped glucomannan gel on a phantom of the chest wall and pleura. However, a spot of ultrasound gel did not generate a long vertical artifact; rather, it generated a short vertical artifact. Based on these results, we suspected that the point of contact of the source with the polypropylene sheet corresponding to the visceral pleura was a key factor influencing the generation, configuration and echo intensity of vertical artifacts [8].

In another study, we used an ultrasound gel spot to imitate the source of the vertical artifact and a block of bacon as a chest wall phantom. As the size of the point of contact between the gel spot on the sheet and the phantom decreased when the sheet was peeled, a vertical artifact was generated and/or extended deeper. Next, objects of different shapes made using gel balls were used to observe the generation of vertical artifacts and compare the echo intensity. For a given shape, the intensity was markedly higher in one model with the point of contact than in the other model with the plane of contact. With the same point or plane of contact, the echo intensity was higher in the taller model. The results obtained from the simple experimental models support the acoustic trap theory [7]. The length and echo intensity of the vertical artifacts depend on the relative size of the channel to the trap volume [7,16]. On top of that, short vertical artifacts, which do not meet the definition of B-lines, may provide clinically significant information about the morphology and structure of the subpleural part [17,18,19].

**Figure 1:**
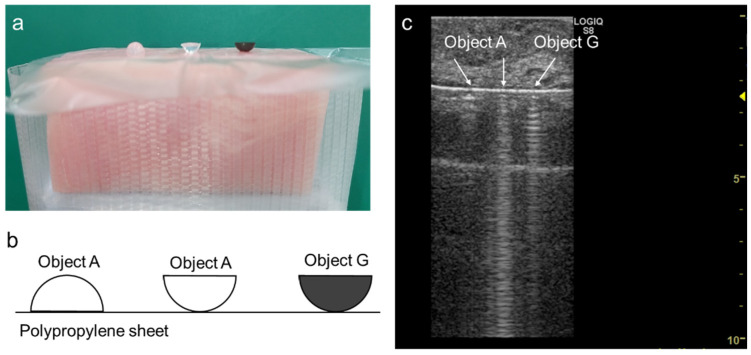
Photograph (**a**) and schematic representation (**b**) of the experimental system and an ultrasound image (**c**) obtained with the system. The object A on the left side generated a short vertical artifact. The object A on the right side generated a long vertical artifact without a qualitative attenuation according to the depth. The object G generated a vertical artifact which was visually attenuated on the deeper side.

In the latter study, we used materials that have similar acoustic properties to water as the sources of vertical artifacts [7]. The model seems to be suitable for considering the mechanism underlying the B-lines in cardiogenic pulmonary edema. If a material with significant sound attenuation is used instead of ultrasound gel or a “pure” gel ball for a given model, it is speculated that the length and intensity of the vertical artifact will be shorter and lower, respectively [7]. This model may be suitable when considering the mechanism underlying B-lines in pulmonary diseases, such as interstitial pneumonia or pulmonary fibrosis. We therefore performed a pilot experiment using a simple model based on this hypothesis. At first, we prepared two tiny hemispherical gel objects of 3.6 mm in diameter that were made of de-aerated water and powdery agar (3%), and a hemispherical gel object of the same size that was made of de-aerated water, powdery agar (3%) and powdery graphite (5%), in which the value of attenuation coefficient was 0.5 dB/cm/MHz. The former and latter object were named “object A” and “object G”, respectively. First, the polypropylene sheet was laid onto a block of bacon as a chest wall phantom, which was coated with a thin layer of ultrasound gel. Two objects A and one object G were then placed onto the sheet through a thin layer of ultrasound gel, as shown in Figure 1a,b. The ultrasound image was obtained by LOGIQ S8 scanner (GE Healthcare) with 9 MHz linear transducer (9L-D). Imaging mode was fundamental B-mode with 6.0 MHz with overall gain and dynamic range were 60 and 72, respectively. Time gain compensation (TGC) was flat as used in clinical examinations. The differences in the configurations of vertical artifacts between the two objects A was the same as that in our previous study [7]. The vertical artifact generated from object G was visually attenuated on the deeper side, in line with the hypothesis (Figure 1c). This pilot experiment and published studies [17,20] indicate that clarification of the relationship between the length and intensity of vertical artifacts and the physical or acoustic composition of the sources may be useful for differentiating a cardiogenic pulmonary edema from a lung disease. This notion needs to be verified in future basic and clinical studies.

## 3. Influence of Machine Settings and the Selection of Transducers on Vertical Artifacts

The optimal machine settings to visualize B-lines are not completely clear, even in the current situation [9]. Many published studies that used B-lines as a metric do not provide information on the machine settings [9]. In this chapter, we explain several machine settings that have a large impact on the configuration of vertical artifacts based on our basic research [8] and clinical experience with some references [21]

### 3.1. Spatial Compound Imaging

**Figure 2:**
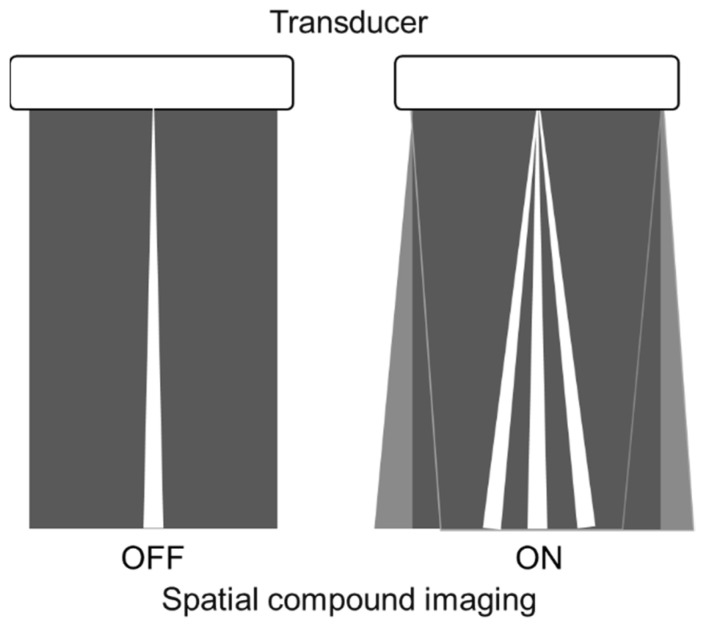
Spatial compound imaging is a method wherein sonographic information is obtained from several different insonation angles and combined to produce a single image.

**Figure 3:**
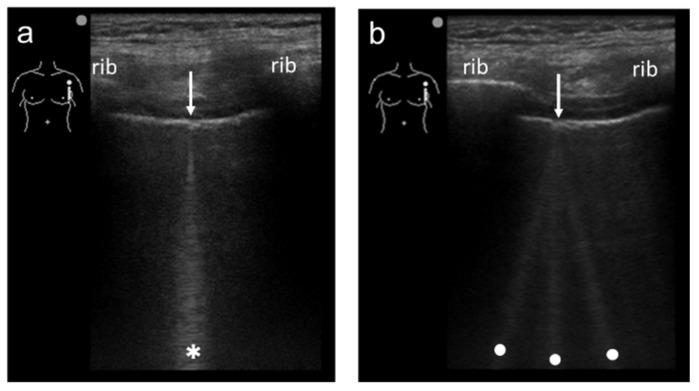
Ultrasound images obtained by MicroMaxx scanner (SonoSite) with a linear transducer without (**a**) and with spatial compound imaging (**b**). A focal point was set as default by the manufacturer, and it is not shown on the screen. With spatial compound imaging enabled, single B-line (asterisk) changes to multiple lines (dots) radiating from the same point (arrows) on the pleural line.

**Figure 4:**
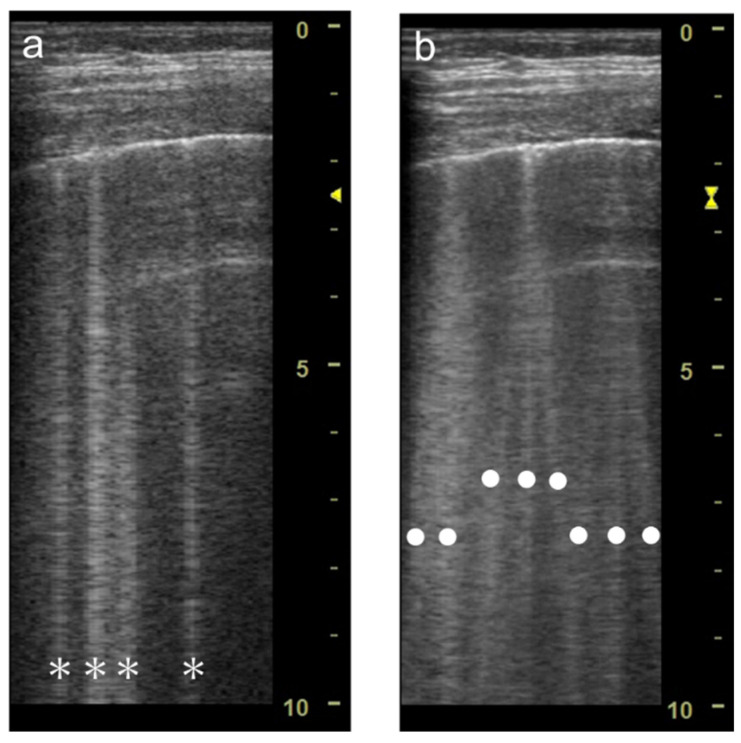
Ultrasound images obtained by LOGIQ V scanner (GE Healthcare) with a linear transducer without (**a**) and with spatial compound imaging (**b**) in cardiogenic pulmonary edema. With spatial compound imaging enabled, each single B-line (asterisks) changes to multiple lines (dots) radiating from the same point on the pleural line.

**Figure 5:**
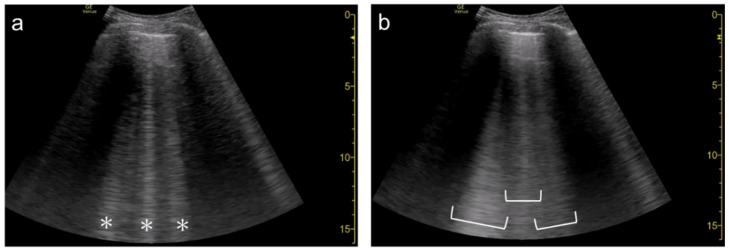
Ultrasound images obtained by Venue scanner (GE Healthcare) with a curvilinear transducer without (**a**) and with spatial compound imaging (**b**) in pulmonary edema. With spatial compound imaging enabled, each B-line (asterisks) becomes divergent with increasing depth, indicating the overlap of multiple lines.

Spatial compound imaging is now available on most ultrasound machines. The main purposes are to improve contrast resolution and to reduce acoustic shadowing. This method acquires three or more multiple images by multiple transmission with different angles, and creates averaging images by overlaying them incoherently [22] (Figure 2). When a linear probe is used with spatial compound imaging enabled, a single B-line changes to multiple lines starting from the same depth of the pleural line [8] (Figure 3 and Figure 4). This is because the vertical artifact generates associated with the scan line. Thus, the vertex of the multiple lines is considered to be a point of the acoustic trap. The angle made by the lines depends on the machine settings. These multiple lines appear to overlap each other with convex probes in some ultrasound machines. In such cases, the resulting “single” line features a divergent appearance with increasing depth [23] (Figure 5).

Many ultrasound machines are now equipped with spatial compound imaging for several applications (e.g., abdominal, breast, thyroid, or vascular ultrasound). If one of the presets for these applications is accidentally selected for the evaluation of B-lines, they may be erroneously counted. To avoid misinterpretation, spatial compound imaging should be set to “off” [8,19,21], or the preset for the lung ultrasound should be selected in advance. In phased array transducers, spatial compound imaging is not mounted; thus, B-lines can be properly evaluated without caution when utilized in addition to echocardiography [9].

### 3.2. Focal Point

**Figure 6:**
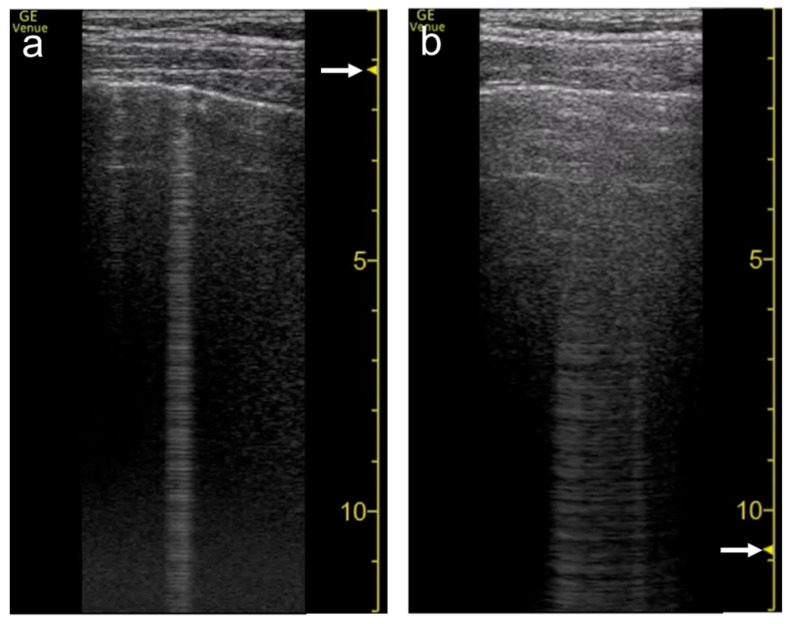
Ultrasound images obtained by Venue scanner (GE Healthcare) with a linear transducer. The B-line becomes wider as the single focal point (arrow) is shifted from the same level as the pleural line (**a**) to a deep level (**b**).

**Figure 7:**
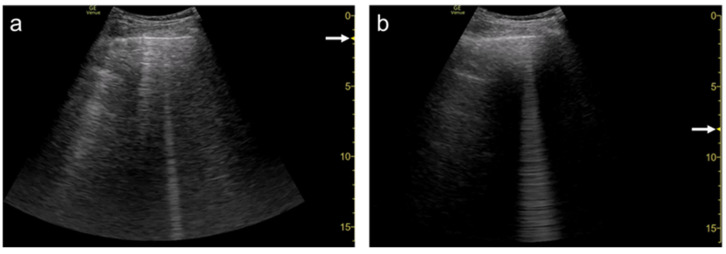
Ultrasound images obtained by Venue scanner (GE Healthcare) with a curvilinear transducer. The B-line becomes wider as the single focal point (arrow) is shifted from the same level as the pleural line (**a**) to a deeper level (**b**).

**Figure 8:**
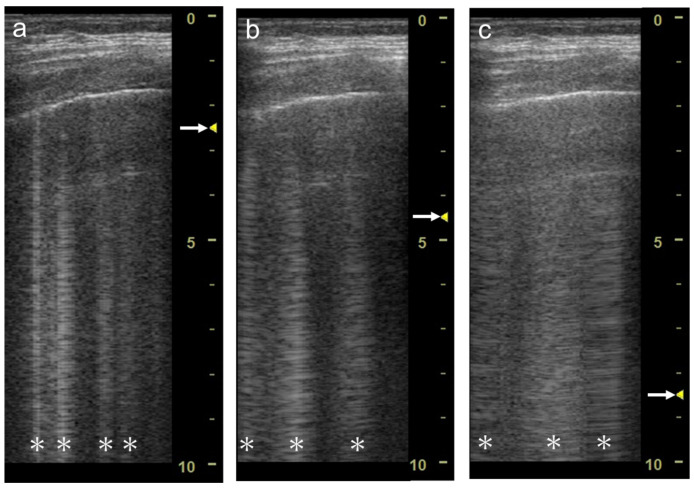
Ultrasound images obtained by LOGIQ V scanner (GE Healthcare) with a linear transducer in cardiogenic pulmonary edema. As the single focal point (arrow) is shifted from a level near the pleural line (**a**) to deeper levels, each B-line becomes wider (**b**), with the B-lines finally overlapping each other (**c**). Asterisks indicate B-lines.

**Figure 9:**
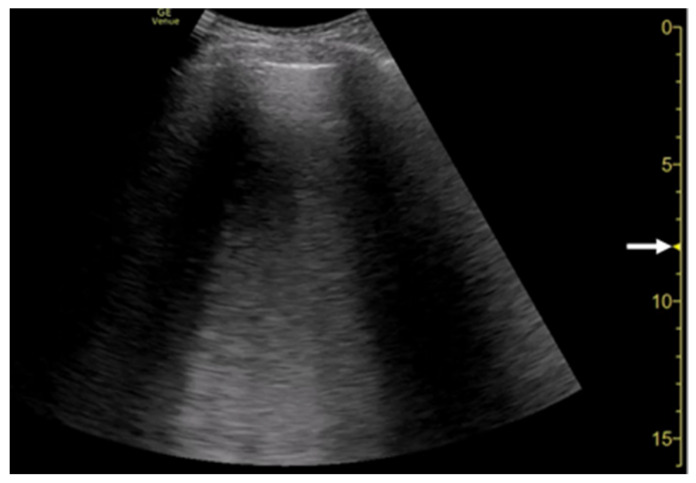
Ultrasound image obtained by Venue scanner (GE Healthcare) with a curvilinear transducer in the same case as Figure 5. As the single focal point (arrow) is shifted to a deeper level, each B-line becomes wider and overlaps each other.

The focal point can also affect the quantification of B-lines. As the single focal point is shifted from the level of the pleural line to deeper levels, the dispersion of B-lines becomes wider during multiple reflections [8] (Figure 6 and Figure 7). As the focal point is shifted to deeper levels, multiple B-lines become wider and can finally overlap each other (Figure 8 and Figure 9). With focused ultrasound, transmission pulse at the focal point hits a small area, whereas the beam width becomes wider in the de-focused area. That means B-lines can be emphasized if the focal point is set to the same depth of the pleural line [16].

In daily practice, confluent B-lines are often observed in cardiogenic pulmonary edema. The confluent B-lines are also called white lung pattern, especially when they cover the intercostal space [24]. However, at present, the confluent B-lines are not precisely defined in the consensus definitions [25]. For consensus, it is recommended that focal point be set at or near the level of the pleural line to ensure “confluence” and accurate quantification or semi-quantification of B-lines [8,9,19,21]. In some portable or hand-held ultrasound machines, the focal point is fixed to a certain level by default and cannot be moved by the user. The level of the focal point should also be considered in the lung ultrasound presets provided by manufacturers [9]. Some ultrasound machines have a function that allows the number of focal points to be changed. In lung ultrasound, B-lines should be evaluated with a single focal point [26].

### 3.3. Frequency

Recent in vitro and in vivo studies have revealed the effect of the frequency on vertical artifacts. Demi et al. [27] and Mento et al. [28] conducted in vitro studies using lung-mimicking phantoms with a multifrequency approach, illustrating how the visualization of vertical artifacts depends on frequency and how native frequency correlates with the geometric characteristic of a bubbly structure. In a clinical study, Mento et al. [20] demonstrated that the quantitative evaluation of vertical artifacts using both a multifrequency analysis and the total intensity may have the potential to discriminate pulmonary fibrosis. Buda et al. [17] examined the visualization and morphological analysis of vertical artifacts by employing two different frequencies. The change of the frequency from 2 to 6 MHz led to the shortening or disappearance of vertical artifacts and this phenomenon is more characteristic of pulmonary fibrosis than cardiogenic pulmonary edema (61% vs. 24% of the examined area, *p* < 0.001). As mentioned above, the visualization and length of the artifacts depend on the frequency; thus, the term “vertical artifacts” has been preferred to “B-lines” in recent studies [29,30].

### 3.4. Selection of Transducers

**Figure 10:**
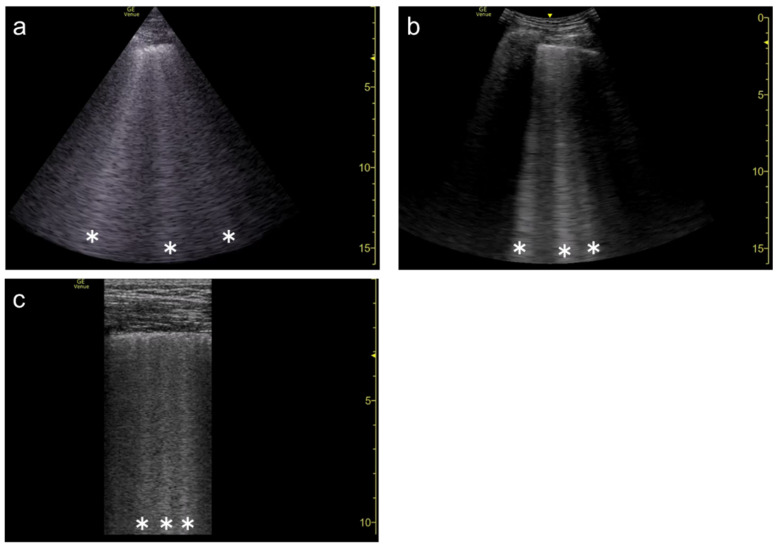
Ultrasound images obtained with Venue scanner (GE Healthcare) with a sector (**a**), curvilinear (**b**), and linear (**c**) transducers in cardiogenic pulmonary edema. Asterisks indicate B-lines.

B-lines are detectable using sector, curvilinear, or linear transducers with low to high central frequencies [1,6]. In sector and curvilinear transducers, multiple B-lines spread radially, whereas in linear probes, multiple B-lines run in parallel (Figure 10).

Many published studies did not mention the presets or used a variety of presets, including cardiac, abdominal, and lung presets set by the manufacturers [9,31,32]; thus, the results should be cautiously interpreted when the performance is compared between the types of transducers. Smit et al. assessed the concordance between a broadband linear transducer (12–4 MHz) and a sector transducer (4–1 MHz) of a handheld ultrasound device in the assessment of lung aeration using B-lines in mechanically ventilated intensive care unit patients. They performed the exams with the lung presets set by the manufacturer in both transducers. There was good concordance between the linear and sector transducers in the assessment; however, a large number of images acquired with the linear probe were of insufficient quality, most likely due to higher attenuation in the subcutaneous tissue layer [33]. The results indicate that a transducer with lower frequency is better for obese and edematous patients.

### 3.5. Simple Educational Materials

Understanding the influence of the above-mentioned machine settings and the selection of transducers on the configuration of vertical artifacts is crucial for planning clinical research, sharing the appropriate use, and providing education in relation to lung ultrasound. However, the necessity has not been fully shared among users. Furthermore, it is not easy to observe the influence of these factors on ‘moving’ vertical artifacts in real patients with or without dyspnea and tachypnea.

A more simplified experimental model is useful for the educational purpose. A motionless stable vertical artifact is easily generated by the model using a glass microscopic plate, which is easily obtainable in each medical facility. We herein demonstrate simple educational materials that help users understand the influence of machine settings on the configuration of vertical artifacts [34]. A diagnostic ultrasound scanner (LOGIQ S8, GE Healthcare) with 9 MHz linear transducer (9L-D) and 3.5 MHz curvilinear transducer (C1-5-D) is used in this demonstration. Fundamental B-mode with 5.0 MHz (linear) and 4.0 MHz (curvilinear) is used. The overall gain and dynamic range are 60 and 72, respectively. TGC is flat as used in clinical examinations.

**Figure 11:**
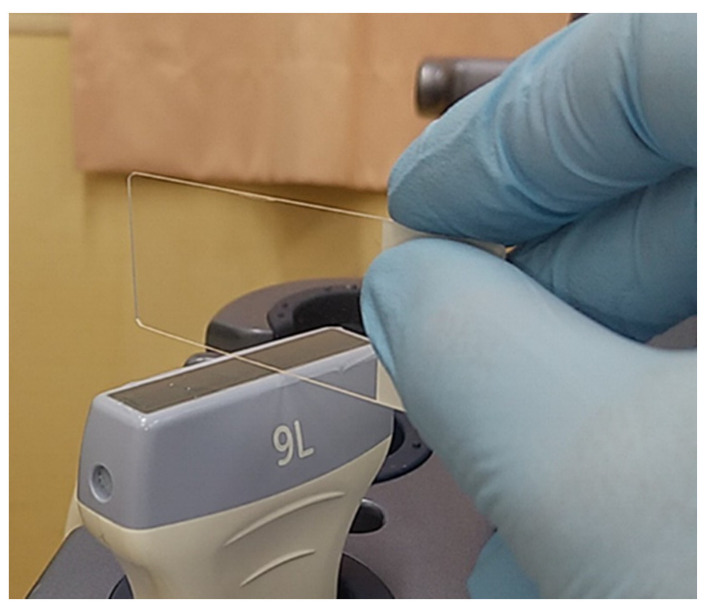
Simple educational materials that help provide an understanding of the influence of machine settings on the configuration of vertical artifacts. A glass microscope slide is placed perpendicularly on the footprint of a linear transducer with a thin layer of ultrasound gel.

As the preset, spatial compound imaging is turned off and the focal zone is set at the shallowest level. A glass microscope slide of 1 mm in thickness (impedance, 12.7 × 10^6^ Pa s/m) is placed perpendicularly on the footprints with a thin layer of ultrasound gel. The thickness of the slide is made parallel to the scan direction (Figure 11). A single clear vertical artifact with a dense cascade of horizontal lines is shown with both linear and curvilinear transducers, while the width of the artifact is wider with the convex transducer than with the linear transducer.

We are convinced that ultrasound beam enters the glass slide. We could observe the change of the configuration or the movement of the vertical artifact when we put ultrasound gel on the upper side of the glass slide and moved the gel with the finger. This fact shows that the ultrasound beam enters the glass slide and reaches the upper side. The following description shows the hypothesis of the generation of the vertical artifact with a dense cascade of horizontal lines. Once the ultrasound pulse wave enters the glass slide, it is trapped with countless reflections and scattering inside the “thin” slide glass. These countless reflections and scattering phenomena act as successive ultrasound sources, with trapped energy radiated to the transducer little by little. The propagation speed of the glass, which is more than 5000 m/s, is another reason for the dense cascade. The other cascade of lines in each image is provided by the reverberation inside the acoustic lens of the transducer rather than the reverberation inside the thin layer of ultrasound gel.

**Figure 12:**
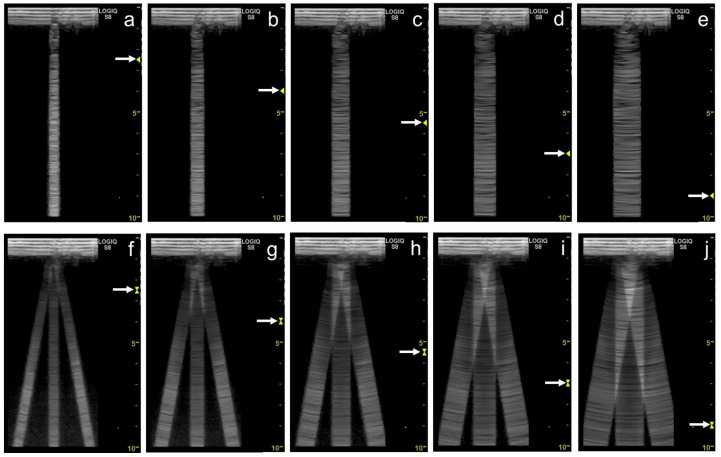
Ultrasound images obtained with the. linear transducer. The vertical artifact becomes wider as the focal point (arrow) is moved to deeper levels (**a**–**e**). When spatial compound imaging is enabled, the single vertical artifact changes to three lines radiating from the same point (**a**,**f**). The three lines become wider as the focal point (arrow) is moved to deeper levels (**f**–**j**).

**Figure 13:**
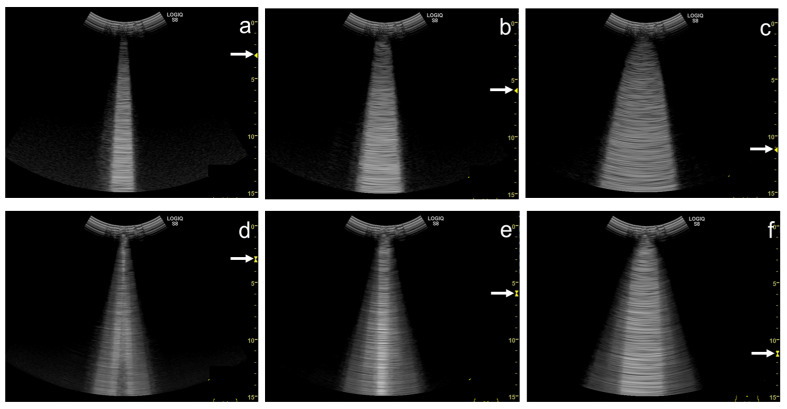
Ultrasound images obtained with the curvilinear transducer. The vertical artifact becomes wider as the focal point (arrow) is moved to deeper levels (**a**–**c**). When spatial compound imaging is enabled, three lines appear to overlap each other (**a**,**d**). The three lines become wider as the focal point (arrow) is moved to deeper levels (**d**–**f**).

As the focal zone is moved from the shallowest level to the deeper levels, the vertical artifact becomes wider with both linear and convex transducers (Figure 12a–e and Figure 13a–c).

When the linear transducer is used with spatial compound imaging enabled, the single vertical artifact changes to three lines radiating from the same point (Figure 12a,f). The three lines become wider as the focal zone is moved to deeper levels (Figure 12 f–j). When the curvilinear transducer is used with spatial compound imaging enabled, the three individual lines appear to overlap each other (Figure 13a,d). These lines become wider as the focal zone is moved to deeper levels (Figure 13d–f).

**Figure 14:**
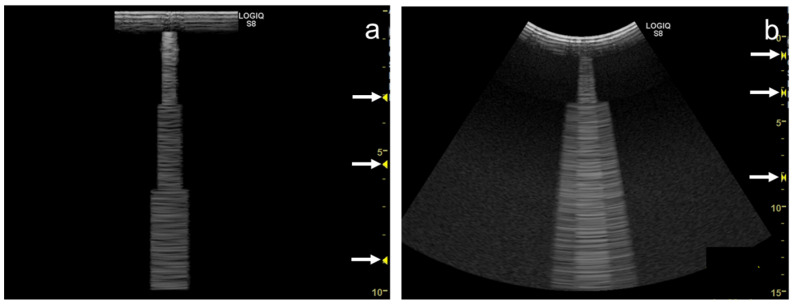
Ultrasound images obtained by a linear transducer (**a**) and a curvilinear transducer (**b**) with multiple focus (arrows), which causes step-wise configuration in a vertical artifact.

Some ultrasound scanners have “multiple focus” in B-mode. It transmits ultrasound pulses in the same direction with a different focal depth, synthesizing these scan-line data (mostly applying focal areas) into one scan line image. As a result, the synthesized image has higher spatial resolution due to the multiple focal points. However, this method causes a “patchy” step-wise configuration in the vertical artifact (Figure 14a,b).

As shown with the series of ultrasound images, the configuration of the vertical artifacts is detrimentally affected by the use of spatial compound imaging, the placement of the focal point to at a deep level, and the use of multiple focus. These simple educational materials tell us the non-negligible impact of the ultrasound machine settings on the morphology of vertical artifacts in lung ultrasound.

## 4. Conclusions

The acoustic trap theory is useful when considering the generation of vertical artifacts in lung ultrasound. Several studies employing basic research support the theory. Published studies with pilot experiments indicate that clarification of the relationship between the length and intensity of the vertical artifacts and the physical or acoustic composition of the sources have potential for differentiating cardiogenic pulmonary edema from lung disease.

In the clinical setting, the configuration of the vertical artifacts is detrimentally affected by the use of spatial compound imaging, the placement of the focal point at a deep level, and the use of multiple focus. Simple educational materials using a glass microscope slide also demonstrate the non-negligible impact of the ultrasound machine settings on the morphology of vertical artifacts. An international consensus needs to be reached on the optimal settings of ultrasound machines, which affect the diagnostic accuracy.

## Data Availability

Not applicable.
